# Health and Health Care for Homeless People in Various Contexts

**DOI:** 10.3390/ijerph15050948

**Published:** 2018-05-10

**Authors:** Stéphanie Vandentorren, Pierre Chauvin

**Affiliations:** 1 INSERM, Sorbonne Université, Institut Pierre Louis d’Épidémiologie et de Santé Publique, Department of Social Epidemiology, 75012 Paris, France; 2 Santé Publique France, French National Public Health Agency, 94410 Saint-Maurice, France; 3 Observatoire du Samusocial de Paris, 75012 Paris, France

Although there is some uncertainty about the exact number of homeless people at any given time in different countries, it is undeniable that this number has steadily and dramatically grown in the recent years [1]. The causes of homelessness are complex: homelessness arises as a result of the interaction between individuals’ history (e.g., poverty, childhood abuse, being in care, family problems, physical illness, loneliness, mental ill-health and/or substance use, adverse life events) and structural/social causes (shortage of low-cost housing, lack of low-skilled jobs, unemployment, immigration, lack of social welfare policies) [2]. 

Surveys conducted over the past three decades have shown that homeless people became more and more diverse in terms of age, sex, ethnicity and family composition [3]. This heterogeneity reflects all the various populations of society, demonstrating the continuum of social situations leading to homelessness. These populations may include adult men and women, elderly and youths, city dwellers and rural residents, migrants and native people, along with families with children, which represents the fastest-growing segment of the homeless population in several countries [4]. This heterogeneity corresponds to a variety of different problems, circumstances and specific needs that this special issue proposes to explore.

There is a wide variability in prevalence of disease according to these sociodemographic profiles, but there is a consistent message that health is significantly poorer for homeless. The effects of homelessness on the deterioration of health are a well-known issue, with an obvious consequence: premature death. Homeless people have much higher rates of physical and mental illness and have a poor access to health care services [5]. They live in unsanitary and insecure conditions. All these factors are amplified by stressful situations and psychiatric disorders that are major causes of concern [6]. This issue illustrates the complexity and intricacy of these risk factors.

Examining differences among subgroups of homeless people allows identifying their specific needs. This information is important for program planning (i.e., drug rehabilitation programs need access to clinicians with expertise in this field although families refugee programs need language and cultural adjustment). Nevertheless, homeless’ basic needs remains being safe, decent housing and income resources. Homeless people typically need, at best, social and health care services that would be easily accessible and fully integrated [7]. Despite the progress of homelessness research, which has clearly demonstrated that homelessness is indeed linked to poverty, solutions are more far-off than ever as the housing and income crisis grows, as socioeconomic inequalities are worsening, as welfare cuts left an increasing number of people on the way side. Actions to prevent and respond to homelessness across the life course are essential, such as reducing the risk of homelessness to children and young people to strengthen their life chances; enabling working age adults to enjoy social, economic and cultural participation in society and breaking the cycle of housing by addressing mental health problems. 

## References

[1] Schlay A., Rossi P. (1992). Social sciences research and contemporary studies of homelessness. Annu. Rev. Social..

[2] Shinn M. (2010). Homelessness, poverty and social exclusion in the United States and Europe. Eur. J. Homelessness.

[3] Grant R., Gracy D., Goldsmith G., Shapiro A., Redlener I.E. (2013). Twenty-five years of child and family homelessness: Where are we now?. Am. J. Public Health.

[4] Bassuk E.L., Rubin L., Lauriat A.S. (1986). Characteristics of sheltered homeless families. Am. J. Public Health.

[5] Fazel S., Geddes J.R., Kushel M. (2014). The health of homeless people in high-income countries: Descriptive epidemiology, health consequences, and clinical and policy recommendations. Lancet.

[6] Dohrenwend B.P., Levav I., Shrout P.E., Schwartz S., Naveh G., Link B.G., Skodol A.E., Stueve A. (1992). Socioeconomic status and psychiatric disorders: The causation-selection issue. Science.

[7] Hwang S.W., Tolomiczenko G., Kouyoumdjian F.G., Garner R.E. (2005). Interventions to improve the health of the homeless: A systematic review. Am. J. Prev. Med..

